# Pulmonary rehabilitation in Lebanon “What do we have”? A national survey among chest physicians

**DOI:** 10.1371/journal.pone.0254419

**Published:** 2021-07-13

**Authors:** Rebecca Farah, Wim Groot, Milena Pavlova

**Affiliations:** 1 Department of Health Services Research, CAPHRI, Maastricht University Medical Center, Faculty of Health, Medicine and Life Sciences, Maastricht University, Maastricht, The Netherlands; 2 Department of Rehabilitation and Physical Therapy (Group A), Chirec-Delta Hospital, Brussels, Belgium; 3 Department of Rehabilitation and Physiotherapy, Bellevue Medical Center, Mansourieh el Metn, Lebanon; University of Rouen-Normandie, FRANCE

## Abstract

**Background:**

Pulmonary rehabilitation (PR) is not very often used by physicians in Lebanon despite evidence on its positive effects on health-related quality of life.

**Aim:**

This study assesses the knowledge, attitudes and practices of PR among physicians in Lebanon. In addition, the study identifies the main barriers to access to PR according to chest physicians. Insight into these issues will help to increase awareness about the need for PR programs and can contribute to designing such programs in the country.

**Methods:**

A survey was conducted during the regional conference of the Lebanese Pulmonary Society. One week after the initial survey, the survey questionnaire was sent by email to all chest physicians who were registered with the Lebanese Pulmonary Society but did not attend the conference. A 25-item questionnaire was used to collect information on PR.

**Results:**

Responses were analyzed using descriptive statistics. The response rate was 40%. Results show that only one-third of Lebanese chest physicians have good knowledge about the nature and multidisciplinary content of PR. Physicians generally support the current “Pulmonary Rehabilitation Program” in Beirut. Key barriers found are the lack of referral, lack of motivation by patients due to their health, cost of care and lack of qualified health care specialists in Lebanon.

**Conclusion:**

Absence of awareness and education about PR among healthcare providers plays an important role in increasing access to the “Pulmonary Rehabilitation Program”. Awareness campaigns and education for physicians, health care professionals and patients should be considered to increase PR in the country.

## Introduction

Pulmonary rehabilitation (PR) is recommended by health professionals and physicians around the world as an essential component of care in the management of respiratory diseases [1–5]. Evidence exists in support of the effectiveness of PR in other respiratory disorders, such as pulmonary fibrosis, pulmonary hypertension and others [6]. Referring to the American Thoracic Society (ATS) and the European Respiratory Society (ERS) viewpoint, “the optimal treatment of individuals with chronic respiratory disease requires combining non-pharmacologic and pharmacologic therapies” [7–10]. Despite the scientific evidence on the benefits, PR is still not very often used by physicians in Lebanon.

Lebanon has a high prevalence of respiratory diseases (asthma, chronic bronchitis and tuberculosis) and pulmonary cancer, according to WHO and the Ministry of Public Health [11]. Despite this, there is only one PR program, which is offered in a hospital in Beirut. This program is composed of 20 sessions, with a frequency of twice a week, over a period of 10 weeks. Each session includes 60 minutes of training consisting of coached physical exercise and a 20 minutes educational intervention. To access this program, patients need a referral from their physician (pulmonologist or general practitioner). However, there is no standard protocol to refer patients to PR. Whether the participation in the PR program is covered by the patients’ insurance depends on the insurance taken by the patient. Additionally, the costs of the program are perceived as high and are not covered by the National Social Security Fund (NSSF) in Lebanon.

In addition to the lack of PR coverage, there is a general lack of awareness about preemptive medical measures like rehabilitation treatments in the Lebanese health care system. As a result, PR is still not routinely recommended by chest physicians. It could thus be presumed that there are barriers to access to rehabilitation treatment, which are related to the patients’ ability to cover the costs and the physicians’ attitude and practice of referrals.

The objective of this study is to identify the knowledge, attitudes and practices (KAP) of Lebanese chest physicians about PR. In particular, this study assesses perceptions about discharge treatment recommendations for patients with respiratory diseases in Lebanon as well as barriers to access and referrals to PR. This study is descriptive and is based on the widely applied KAP framework [12]. We believe that more insight into the KAP of the providers can help to increase awareness about the need for PR programs in Lebanon and can contribute to designing these programs in the country. This is important as the country is facing a high rate of pulmonary diseases (number one cause of death) while there is a lack of funding to ensure equitable access for the entire population in need of PR. Our study can contribute to a broader discussion on implementing effective PR programs in Lebanon and other low- and middle-income countries.

## Methods

This study was performed according to the Declaration of Helsinki, ICH-GCP. The study was approved by the Lebanese Pulmonary Society (LPS) and was reviewed by the Ethics Committee of Maastricht University. The study has a quantitative cross-sectional design. It was carried out among Lebanese chest physicians registered with the LPS. The study used a KAP questionnaire about PR in Lebanon. With the collaboration and with the approval of the LPS, a survey was conducted during the regional conference of the LPS held in September 2018, in Beqaa, Lebanon. The LPS has 180 members who are pulmonologists working in Lebanon.

The participants were Lebanese chest physicians, who attended the regional LPS conference. In total, 51 members of the society filled in the survey questionnaire during the conference. There was no selection of respondents. All conference participants, regardless of their ethnicity, religion and political orientation, could take the survey. The participants did not receive any incentive to take part in the study. The survey was advocated by the President of the LPS during the conference and all physicians at the conference, were invited to participate by filling in the questionnaire. The questionnaire was distributed by hand by the LPS Board after a presentation on the aim and purpose of the survey. The participants filled in the questionnaire during the conference. the filled-in questionnaire were collected by the secretary of the LPS during the two days of the conference. We note that the survey was completed by chest physicians present and registered at the annual conference of the LPS. The respondents could fill in the questionnaire in any way they liked, including a quiet place with enough privacy.

Ten days after the conference, the online version of the questionnaire was sent by the President of the LPS by email to the chest physicians who were registered with the LPS but did not attend the conference. Two weeks later, a gentle reminder was sent to those physicians in order to gather more responses.

The questionnaire was based on the KAP framework of the World Health Organization [12] and contained 25 questions. Before the survey, the questions were discussed with professionals in this field and pre-tested. S1 Appendix shows the complete questionnaire. The questionnaire was divided in 5 parts about PR in general:

- Part 1 covered questions on knowledge of physicians about PR
- Part 2 explored the attitudes of chest physicians on PR
- Part 3 assessed the daily practices of respiratory physicians on PR
- Part 4 investigated the barriers faced by physicians when refereeing patients
- Part 5 investigated the socio-demographic data of the responders.

The questionnaire was anonymous. Written informed consent was obtained at the beginning of the questionnaire from each participant who attended the conference and from participants who filled in the online survey questionnaire.

Data entry, data cleaning and statistical analysis were done using the statistical software package SPSS. The analysis consisted of descriptive statistics only, namely frequencies, mean and standard deviation for each response variable. In addition, we investigated the association between the responses to the KAP questions (dependent variables) and the socio-demographic factors of the respondents, such as age, place of graduation (independent variables). For this purpose, we used regression analysis for ordinal data given the ordinal nature of the dependent variables. The results of these regressions were not significant, i.e. no statistically significant association between respondents’ answers and respondents’ characteristics was found. We therefore decided not to report these results in this paper.

## Results

In total, 62 questionnaires were gathered among the 180 members of the LPS (51 during the conference and 11 after distributing the online version). However, 21 physicians listed as LPS members had no (correct) email address and could not be contacted. Therefore, they were disregarded. Thus, the overall response rate was 34%. Results are represented in the form of tables.

### Socio-demographic results

The socio-demographic results in Table 1 show that about half of the participants (53.2%) graduated in Lebanon. Participants were 72.6% males and 27.4% females. In total, 37.1% of the participants were aged between 40–55 years. In addition, 75.8% of the participants practiced in an urban area in Lebanon.

**Table 1:** Socio-demographic features of the participants, N = 62.

Variable	Categories	n(%)	Mean
			SD
Age	<35	5 (8.1%)	3.10
	35–40	11 (17.7%)	1.036
	40–55	23 (37.1%)	
	55–65	19 (30.6%)	
	>65	4 (6.5%)	
Gender	Male	45 (72.6%)	1.73
	Female	17 (27.4%)	0.450
Education	Lebanon	33 (53.2%)	-
	US	5 (8.1%)	
	EU	19 (30.6%)	
	Others	5 (8.1%)	
Work location	Rural	15 (24.2%)	1.76
	Urban	47 (75.8%)	0.432
Place of graduation	Lebanon	33 (53.2%)	-
	US	5 (8.1%)	
	EU	19 (30.7%)	
	Others	5 (8.1%)	

### Knowledge about PR

Table 2 presents the results on the level of knowledge about PR. The table shows that one-third (33.9%) of the participants said they had good knowledge of PR and 11.3% said they had very poor knowledge of PR. Regarding the content of the program, 29% of participants indicated that they had good knowledge of the “multidisciplinary components” of PR but 11.3% indicated that they had very poor knowledge of the multidisciplinary PR components.

**Table 2:** Knowledge of chest physicians about pulmonary rehabilitation (PR), N = 62.

Variable	Categories	n (%)	Mean
			SD
Level of knowledge	Very poor	7 (11.3%)	3.31
	Poor	8 (12.9%)	1.223
	Medium	16 (25.8%)	
	Good	21 (33.9%)	
	Excellent	10 (16.1%)	
Content of knowledge of PR	Very poor	7 (11.3%)	3.18
	Poor	11 (17.7%)	1.222
	Medium	17 (27.4%)	
	Good	18 (29.0%)	
	Excellent	9 (14.5%)	

### Attitude towards PR

Table 3 shows the results on attitudes towards PR. Half of the chest physicians in our study (48.4%) agreed that a COPD patient who was stable could be enrolled into PR. Only one-third (35.5%) of the participants thought that PR in Lebanon was not effective. More than half of respondents (53.2%) thought that access to an outpatient PR center had an added value. Overall, 38.7% of the chest physicians in our sample strongly agreed that quality of treatment increased if a patient enrolled in PR.

**Table 3:** Attitude of chest physicians towards pulmonary rehabilitation (PR), N = 62.

Variable	Categories	n (%)	Mean
			SD
Do you think that COPD patient who is stable could be enrolled into a PR?	Strongly agree	19 (30.0%)	2.03
	Agree	30 (48.4%)	1.008
	Neutral	8 (12.9%)	
	Disagree	2 (3.2%)	
	Strongly disagree	3 (4.8%)	
Do you think that PR in Lebanon is effective?	Strongly agree	5 (8.1%)	3.21
	Agree	12 (19.4%)	1.161
	Neutral	15 (24.2%)	
	Disagree	22 (35.5%)	
	Strongly disagree	8 (12.9%)	
Do you consider that access for an outpatient center is an added value in the country?	Strongly agree	14 (22.6%)	2.13
	Agree	33 (53.2%)	0.949
	Neutral	11 (17.7%)	
	Disagree	1 (1.6%)	
	Strongly disagree	3 (4.8%)	
Quality of your treatment will be increased if your patients will be enrolled in a PR?	Strongly agree	24 (38.7%)	1.76
	Agree	31 (50.0%)	0.761
	Neutral	6 (9.6%)	
	Disagree	0 (0%)	
	Strongly disagree	1 (1.6%)	

### Practice of PR referrals

Table 4 shows the results on the referrals to PR by chest physicians. The table shows that half of the physicians in our study would refer patients “starting in a hospital setting” to adhere to PR. For 59 participants out of 62, a COPD patient was the first type of patient they thought of to enroll in PR.

**Table 4:** Practice of chest physicians regarding pulmonary rehabilitation (PR), N = 62.

Variable	Categories	n(%)	Mean
			SD
When would you refer a patient to start a PR?	Starting in hospital settings	31 (50.0%)	-
	Directly after hospital discharge	16 (25.8%)	
	4 weeks or more after discharge	13 (21.0%)	
	You would not refer	2 (3.2%)	
What kind of patients do you consider suitable for rehabilitation after discharge from hospital?	COPD	59 (95.0%)	-
	CF	44 (70.8%)	
	Bronchiectasis	44 (70.8%)	
	Post thoracic surgery	44 (70.8%)	
	IPF	43 (69.4%)	
Is it difficult to refer patients to PR in the country?	Yes	60 (96.8%)	1.03
	No	2 (3.2%)	0.178
Who should take initiative to initiate PR in the country?	Insurance companies	54 (87.1%)	-
	Professional health givers	53 (85.3%)	
	Physicians	55 (88.6%)	
	Policy providers	50 (80.5%)	
How frequently do you send COPD patients to PR?	3–5 times per month	10 (16.1%)	-
	3–5 times per week	8 (12.9%)	
	1–2 times per month	12 (19.4%)	
	Once every 6 months	10 (16.1%)	
	Never	22 (35.5%)	
After discharge, physicians asked patients to:	To do nothing, to be at rest	2 (3.3%)	-
	To exercise a bit	25 (41.0%)	
	To go to fitness club	9 (14.8%)	
	To take it easy	25 (41.0%)	
	To start a rehab program	1 (1.0%)	

Results show that 96.8% of chest physicians in the study found it difficult to refer patients to PR in Lebanon. In addition, 88.6% of the physicians stated that actions should be taken by physicians to develop a PR program, 87.1% agreed that insurance companies should take such initiatives, 85.3% agreed that such initiatives should be taken by professional health care providers (physical therapist, nurses) and 80.5% supported initiatives by policy makers.

More than one-third (35.5%) stated that they had never referred a patient to a PR program in their daily practice and 19.4% specified that they sent patients to PR 1–2 times per month. After discharging the patient from hospital, 41% of physicians in the study asked their patients to exercise a bit or to participate in a rehab program. At the same time, 41% of the physicians in the study asked their patients to take it easy and 3.3% recommended their patients “to get bedrest”.

### Barriers to PR

Table 5 shows that nearly all Lebanese chest physicians in our study (91.9%) face barriers to refer patients to PR. The major barriers faced were: the location of the center for PR (93.8%) and a lack of motivation by patients due to their health condition (92.7%). For 84.4% of the physicians surveyed, the cost of care was a key barrier and for 85.4%, the lack of specialists was a key limitation for PR. In total, 85.4% of participants in the study stated that the absence of awareness and education about the program were important barriers.

**Table 5:** Kind of barriers facing by respondents to pulmonary rehabilitation (PR), N = 62.

Variable	Categories	n(%)	Mean
			SD
If Lebanese physicians are facing barriers to refer patients to the PR	Yes	57 (91.9%)	1.10
	No	5 (8.1%)	0.298
Lack of specialist, knowledge	No	9 (14.6%)	1.85
	Yes	53 (85.4%)	0.354
Lack of motivation	No	4 (7.3%)	1.92
	Yes	58 (92.7%)	0.26
Absence of awareness and education	No	9 (14.6%)	1.85
	Yes	53 (85.4%)	0.354
High cost of care	No	10 (15.6%)	1.84
	Yes	52 (84.4%)	0.365
Location of the center	No	4 (6.3%)	1.93
	Yes	59 (93.8%)	0.240
All of the above	No	26 (41.7%)	1.58
	Yes	36 (58.3%)	0.490

### Support to PR

Results shown in Table 6 indicate that 93.5% of the Lebanese chest physicians in the study supported an “outpatient” PR program, 83.8% of them supported an “inpatient” PR program and 80.6% supported “home-based” pulmonary tele-rehabilitation”.

**Table 6:** Support from physicians to different types of pulmonary rehabilitation (PR), N = 62.

Variable	Categories	n(%)	Mean
			SD
Support for inpatient PR	Yes	52 (83.87%)	1.18
	No	10 (16.13%)	0.385
Support for outpatient PR	Yes	58 (93.55%)	1.06
	No	4 (6.45%)	0.248
Support for home-based pulmonary tele-rehabilitation	Yes	50 (80.65%)	1.19
	No	12 (19.35%)	0.398

The results of the regression analysis did not indicate any statistically significant association between the responses to questions related to knowledge, attitude and practice of PR and the socio-demographic factors of the respondents, like age, place of graduation.

## Discussion

This is the first study, which has assessed PR in Lebanon. Our study has investigated the knowledge about PR in general, attitude toward PR and practice of referring patients to PR among chest physicians in Lebanon. Barriers faced by chest physicians have been highlighted as well. Based on a 25 items-questionnaire, data were collected and analyzed descriptively.

The results showed that knowledge about PR among the physicians in our study is insufficient. As shown by previous studies, PR programs with its complementary components, such as an education session, a smoking cessation program, pharmacological treatment, diet and psychological support, are crucial for rehabilitation [13–16]. Such multidisciplinary PR programs are indispensable to reduce the burden of respiratory diseases and to offer appropriate treatment as recommended by international guidelines [17]. The lack of knowledge on PR among chest physicians in Lebanon that we find in this study, corresponds to findings for other countries [18, 19]. The ATS/ERS statement for enhancing the implementation, use and delivery of PR, recommends formal training in PR for any healthcare professionals involved in the care of people with COPD. In addition, the ATS/ERS recommended to promote PR through social media and patient support associations as a means of increasing knowledge and awareness of the benefits, process and outcomes of PR. The low utilization of PR in Lebanon is linked, among others, to the lack of patient referrals [19–21]. This weakness in the referral practice could be due to a lack of awareness among physicians about the nature and benefits of PR. The approval and application of guidelines to encourage PR, may help to improve awareness and acceptability of PR, and may increase the knowledge of expected PR outcomes, according to one study conducted in Australia [22].

Attitudes towards PR among Lebanese chest physicians were overall positive. Physicians in our study agree that PR will have an added value to the care for patients and strongly approved that quality of treatment will increase for patients enrolled in PR. In general, Lebanese chest physicians in our study support PR in all kinds of settings: inpatient, outpatient and home-based tele-rehabilitation.

The analysis of the practice of Lebanese chest physicians showed that a large proportion was not aware of the existence of a PR program in the country and highly recommended “the need of implementing the first program”. It is therefore important to promote the available PR program(s) among both patients and physicians.

The low utilization of PR is not a problem in Lebanon only. According to research conducted in Canada, less than 5% of eligible patients receive PR annually [23]. In Lebanon and elsewhere, health care providers are facing barriers to send patients to PR [24, 25]. Identifying these barriers is essential for the development and provision of PR in particular in poor resource areas of the world.

Our study has allowed us to identify and highlight key barriers to access to PR in Lebanon. The major barriers faced are: the difficult localization of the centers, the lack of motivation by patients, the cost of care and the lack of qualified health care specialists. Also, as indicated by our results, the absence of awareness and education about PR programs are important barriers. In addition, we also need to acknowledge the type of health care system in Lebanon, which is more private than public. Such a health care system does not facilitate adherence to treatment as it does not assure equal access to care. A major fact to add is that the NSSF and the government in Lebanon do not routinely finance PR in health centers. Therefore, the financial barriers to PR in Lebanon are substantial and equity of access to PR care is not guaranteed.

Results of other studies have shown similar barriers to PR in other countries: poor referral rates, lack of perceived benefits of the program by physicians and patients, limited availability of and access to services, insufficient programs and an inadequate number of qualified health professionals and patients-related factors [26–30]. According to the literature on PR “a significant barrier to the effective utilization of pulmonary rehabilitation” in the community is the lack of patient referrals, likely due to a lack of awareness among healthcare providers of the nature and benefits of this intervention. Education of professional healthcare givers and promoting awareness among them is highly necessary to increase access, knowledge and use of PR [2].

It is recommended to encourage the implementation of PR programs in Lebanon to increase access to PR for patients suffering from respiratory diseases. PR needs to be established in Physical Therapy and Rehabilitation departments and needs to be part of the conventional treatment for COPD and non-COPD patients as suggested by the guidelines of ERS/ATS. To increase patients’ quality of life, to reduce readmission and future exacerbations in COPD patients, PR must be applied as a tailored program. PR needs to start as early as possible during the first phase of hospitalization and above all in the ICU and should be available when needed until the discharge of the patient as recommended by internationals experts [2].

In Lebanon, a second PR program is planned in the Mount-Lebanon area. This will facilitate access to the inhabitants of this area in addition to the first PR established in the West of Beirut. The location of this new PR is strategic; about 10 km east of Beirut and in a green area surrounded by a green hilltop of pines trees overseeing the capital. This PR will be accessible to patients of rural villages in Mount-Lebanon and patients from Beirut’s urban area, which is very close by.

### What kind of recommendation for future PR implementation?

To overcome local barriers (location, cost, lack of time etc.) in Lebanon, home-based PR or/and tele-rehabilitation might be suggested. Home base programs have been found to be effective. One study conducted by Grosbois and all [31] on COPD and asthma patients suggested that home-based PR improved exercise tolerance and quality of life in severe asthma. In addition, the effect of home-based PR in severe asthma and COPD is maintained up to 12 months after PR program. Another study shows that home-based PR is an efficient non-pharmacological therapy that improves exercise tolerance and HRQoL in COPD patients [32]. Home-based PR is being prescribed more widely than hospital-based rehab due to the lower cost and ease of caregiver burden.

In addition, tele-rehabilitation is an emerging rehab measure where patients with COPD or suffering from respiratory diseases exercise at home, while experts from the tertiary care centers monitor the rehab sessions remotely. In general, we recommend to focus more on multiple ways of pulmonary tele-rehabilitation delivery in Lebanon. Aside from home-based PR and tele-rehabilitation, this could include web-based tele-rehabilitation, center-based tele-rehabilitation and home-based tele-rehabilitation. Studies have found that home-based tele-rehabilitation can achieve clinically important gains in health-related quality of life [33–35].

To close the knowledge gap on PR in Lebanon, we suggest for further research to repeat the study among primary care providers. A big challenge in Lebanon is to change not only physicians’ referral behavior but also patients’ unhealthy behavior including well-established habits like lack of physical activity and a high prevalence of smoking waterpipe. This point deserves consideration, although it was not studied in this paper, but we would like to emphasize its importance for the implementation of PR in poor resource areas with high degree of air pollution.

The strength of our study is that it investigates PR in Lebanon targeting all chest physicians. No study has been conducted before on this topic in Lebanon. However, the study has several limitations that must be underlined. First, some participants were not aware of the only PR program offered in the country. Many of them thought that there was no PR program yet. This questions their ability to adequately reflect on the situation. Another limitation is our questionnaire, which was designed in English language only. It could have been translated in Arabic and French to better facilitate the comprehension by all chest physicians. Nevertheless, all respondents had a good command of English language. The respondents could fill in the questionnaire in a quiet place and with enough privacy but we acknowledge that the conditions might not have been optimal for some respondents. The last limitation is the fact that there were several missing or incorrect email addresses, which means that some potential respondents could not be contacted. In addition, many physicians contacted did not participate. We do not know why some physicians contacted through email did not respond. But it is known that online surveys usually result in a low response rate.

## Conclusion

This is the first study in Lebanon to investigate KAP among chest physicians on PR. Our study has demonstrated that many chest physicians in Lebanon are not aware of the only PR program in the country. To overcome barriers to access and use PR, international guidelines need to be followed. An imperative change in the access to the Lebanese health care system will be required to cover secondary prevention, such as cost-effective PR programs. Additionally, a challenge is the need to change the knowledge, attitude, and practice of healthcare professionals by making them more aware of the benefits of PR for their patients and encouraging them to refer more often patients to PR.

## Supporting information

S1 Appendix 25 items in the questionnaire. (TIF)
