# Supplementary figures and images for: Pulmonary rehabilitation in Lebanon “What do we have”? A national survey among chest physicians
Source: PLoS One. 2021 Jul 13;16(7):e0254419. doi: 10.1371/journal.pone.0254419 (PMC8277028; doi:10.1371/journal.pone.0254419)

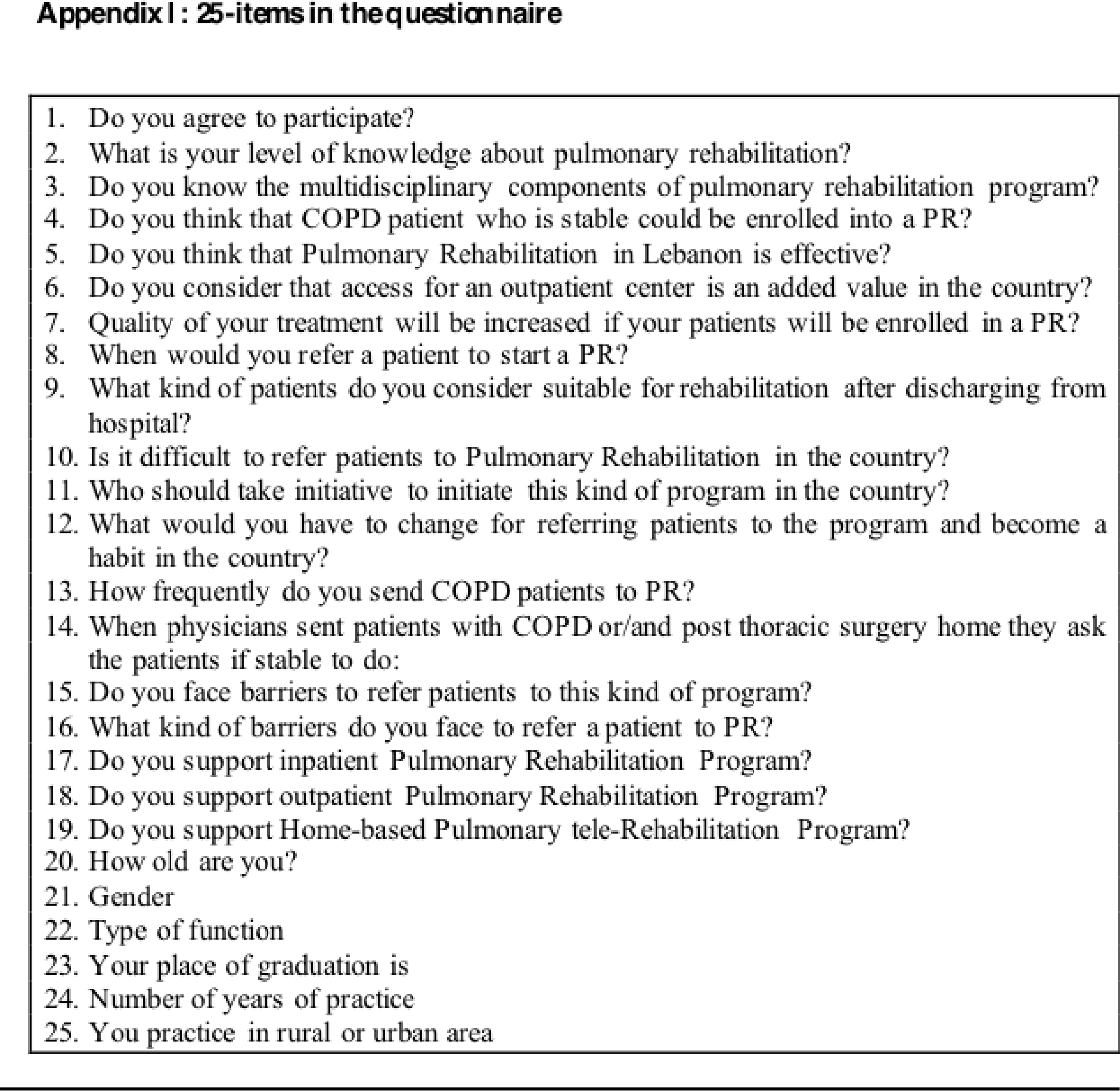

Supplement: S1 Appendix — (TIF) [file pone.0254419.s001.tif]
